# Application of Hybrid External Skeletal Fixation with Bone Tissue Engineering Techniques for Comminuted Fracture of the Proximal Radius in a Dog

**DOI:** 10.3390/ani14233480

**Published:** 2024-12-02

**Authors:** Minji Bae, Byung-Jae Kang, Junhyung Kim

**Affiliations:** 1 Department of Veterinary Medicine, College of Veterinary Medicine and Institute of Veterinary Science, Kangwon National University, Chuncheon 24341, Republic of Korea; mangdi1224@kangwon.ac.kr; 2 Department of Veterinary Clinical Sciences, College of Veterinary Medicine and Research Institute for Veterinary Science, Seoul National University, Seoul 08826, Republic of Korea; bjkang81@snu.ac.kr

**Keywords:** radial head fracture, bone tissue engineering, hybrid ESF, gait analysis, canine

## Abstract

A seven-month-old male Pomeranian presented with left forelimb lameness after a fall. Radiographic assessment confirmed proximal radial head and ulnar comminuted fracture. The initial surgical intervention involved the use of hybrid external skeletal fixation (ESF) to stabilize the radial head, concomitant with the application of a composite of bone morphogenetic protein type 2 (BMP-2)-loaded hydroxyapatite and gelatin microparticles at the fracture site. Although successful radial head healing was achieved, the ESF pinholes caused a defect in the proximal ulnar diaphysis. Subsequently, the ESF was removed, and a locking plate was applied in conjunction with the BMP-2-loaded collagen membrane to correct the radius defect. Clinical follow-up at 4.8 years postoperatively revealed a mildly decreased range of motion of the affected elbow joint, but no clinical symptoms such as lameness. Radiography revealed minimal degenerative changes and a radioulnar synostosis. Computed tomography revealed differences in the leg length and bone density. Gait analysis revealed that the left forelimb had a significant improvement in weight-bearing capacity based on weight distribution–peak vertical force metrics, compared with the right forelimb. Based on clinical outcomes, the combined application of hybrid ESF and bone tissue engineering techniques can be considered a feasible alternative treatment for radial head fractures.

## Simple Summary

This case report follows the recovery of a seven-month-old Pomeranian who had a serious fracture in its forelimb. The patient needed two surgeries to fix the fracture and help the bone heal using advanced methods. First, an external frame was used to keep the bone in place, along with a special material to help the bone grow. Although the bone initially healed well, problems with the frame caused the need for a second surgery. In the second surgery, a plate was added to support the bone, and a new healing material was used. After almost five years, the patient had less movement in the injured leg but did not seem to be in pain. X-rays showed only small changes in the joints, and although CT scans showed differences in leg length and bone density, the patient’s ability to bear weight on the treated leg improved significantly. This case shows that combining these advanced techniques can be a good approach for treating complex fractures in veterinary practice.

## 1. Introduction

The prevailing fracture sites of small and toy breed dogs regarding age and body size are as follows: radius/ulna, tibia, femur, and humerus [1,2,3]. The radius and ulna are relatively thin and long in small and toy breed dogs, making fractures more common when excessive pressure is applied to their forelimbs, particularly during activities such as jumping, or following external impacts such as falls. Most radial and ulnar fractures occur in the distal region, whereas fractures in the proximal region are less common. Proximal radial fractures are rare and challenging to access surgically because of anatomical constraints [4]. Although internal fixation provides greater stability in typical proximal radial transverse fractures, placement of implants onto the radial head becomes challenging in cases of more comminuted radial head and neck fractures. In such situations, external skeletal fixation (ESF) devices can be employed as a surgical approach [3,5,6].

ESF offers the advantage of minimizing soft tissue damage, making it an effective option for fractures in regions with relatively little soft tissue [7,8,9,10]. Additionally, the surgeon can apply a bilateral or biplane frame to further stabilize the fracture. The hybrid ESF technique, which combines both linear and circular external fixation components, is commonly recommended for fractures involving the juxta-articular bone segments near the joints. This is particularly valuable in cases where the application of plate screws is challenging [7]. Furthermore, in cases of complex fractures affecting the radial head and neck, the use of a hybrid ESF may be indicated to float the radial head and maintain its position, utilizing a linear ESF that secures the olecranon proximally and the radius distally, thereby allowing the radial head to be floated and stabilized by the surrounding soft tissues [4].

In recent years, extensive research has been conducted in the fields of tissue engineering and regenerative medicine to identify methods for enhancing bone healing [11,12]. Bone tissue engineering (BTE) focuses on the development of innovative biomedical techniques to treat skeletal injuries and is a promising treatment method for animals with fractures. BTE strategies involve the application of bone grafts to a scaffold, which are subsequently implanted in vivo to target bone defects; however, its long-term outcomes have not been sufficiently characterized yet.

To the authors’ knowledge, there are currently no documented cases in the veterinary field that apply hybrid ESF in conjunction with BTE for fractures of the proximal radius, and no cases have confirmed the bone status through long-term follow-up after the application of BTE. This paper reports the bone status and gait of a small breed dog treated with hybrid ESF using BTE techniques 4.8 years after the procedure.

## 2. Case Description

**Figure 1:**
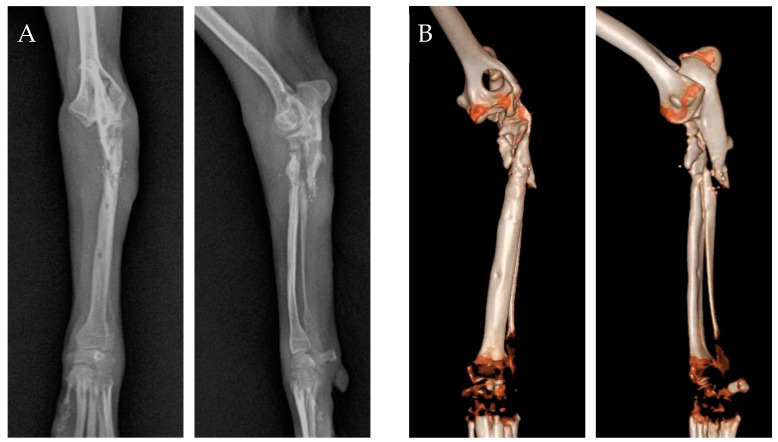
(**A**) Preoperative radiography image. (**B**) Preoperative computed tomography reconstruction image.

A 7-month-old, 3.3 kg, intact male Pomeranian suffered a complex fracture of the left proximal radius and ulna after falling. A tie-in IM pin was placed to fix the fracture at a private veterinary clinic; however, the fixation failed, and the fracture recurred. At referral, the patient could not weight-bear on the left forelimb during the standing physical examination. For subjective evaluation, the clinical lameness scores were used, which were based on six factors such as lameness score, range of motion (ROM), and pain, with each factor evaluated on a scale of 1 to 4 or 5 points (total score range: 6 to 27) [13]. Assessment using the clinical lameness score criteria revealed 24 points out of 27 points. Radiographs revealed poor bone healing due to the previous pin fixation and a comminuted fracture of the left proximal radius and ulna (Figure 1A). As the fracture was near the radial head, the bone structure was not clearly visible on radiography, necessitating a computed tomography (CT) scan for accurate fracture assessment and appropriate surgical planning. CT revealed a proximal non-reducible radial head fracture, which was fractured into three proximal fragments (6.5 mm × 5.9 mm, 7.5 mm × 2.9 mm, 10.4 mm × 4.1 mm), along with a proximal ulnar fracture (Figure 1B). Articular surface damage was also observed. Based on comprehensive examination, it was determined that stabilizing the radial head was essential to preserve the affected forelimb. Due to the radial head’s non-reducible fracture, direct stabilization through implant fixation was not feasible. Therefore, as a surgical treatment strategy, it was decided to apply a type II hybrid ESF, combining a bilateral uniplanar linear system with an Ilizarov-type circular system, to create space for the placement of two screws on the radial head.

### 2.1. Surgical Treatment I

After obtaining a CT image of the left forelimb, a 3D anatomical model was fabricated for preoperative planning and surgical rehearsal using an ESF device.

**Figure 2:**
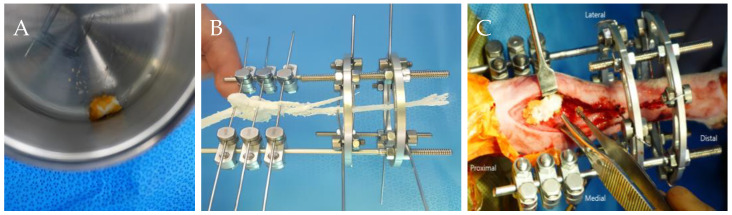
Intraoperative images taken during the first surgery. (**A**) Gelatin microparticle (GMP) loaded with BMP-2; (**B**) simulation of the external skeletal fixation (ESF) device on a 3D-printed model; (**C**) insertion of the BMP-2 loaded HA and GMP mixture at the radius defect site.

The first surgical treatment was performed one week after referral (Figure 2A–C). The patient was premedicated with 0.2 mg/kg of intravenous (IV) midazolam (midazolam, Inj^®^; Bukwang Pharm, Seoul, Republic of Korea). Anesthesia was induced with 6 mg/kg of IV propofol (Anepol, Inj^®^; Hana Pharm, Seoul, Republic of Korea) and maintained with inhaled isoflurane (Ifran; Hana Pharm, Seoul, Republic of Korea) in oxygen. Cefazolin (Cefazolin, Inj^®^; Chong Kun Dang Pharm, Seoul, Republic of Korea) a prophylactic antimicrobial agent, was administered before surgery and intraoperatively at an initial dose of 25 mg/kg IV, with repeat doses every 90 min throughout the procedure. The dog was positioned in lateral recumbency, with the affected limb placed in the uppermost position. The skin was aseptically prepared for surgery. After palpating the olecranon, a craniomedial skin incision was made at the proximal radius. Proximal radial defects were observed, and a predesigned hybrid ESF (IMEX™ Veterinary, Inc., Longview, TX, USA) was applied, combining type II linear ESF at the proximal site of the fracture and two circular ESFs at the distal site of the fracture. The linear ESF was equipped with six connecting clamps and fixation pins in the form of sequential wires (1.0 mm, 0.9 mm, and 1.0 mm) from the most proximal to the distal part of the olecranon. Circular ESFs were implanted using two 0.9 mm k-wires at a distal radius. A bone graft mixture comprising recombinant human bone morphogenetic protein type-2 (rhBMP-2) (0.25 mg; NOVOSIS, Seoul, Republic of Korea) loaded with hydroxyapatite (HA) and gelatin microparticles (GMP) was inserted into the defect site to expedite bone healing. The dosage of rhBMP-2 was determined as described by Massie et al. [14]. After insertion of the bone graft, the muscles and skin layers were closed routinely. Postoperatively, cephalexin (Phalexin capsules, Donghwa Pharm, Seoul, Republic of Korea) was administered at 25 mg/kg q12h PO for 5 days. Postoperative analgesia involved intermittent tramadol 4 mg/kg PO (Tridol, Yuhan Pharm, Seoul, Republic of Korea) as necessary. Meloxicam (Metacam, Boehringer Ingelheim, Seoul, Republic of Korea) was administered at 0.2 mg/kg IV on the day of surgery followed by 0.1 mg/kg PO starting 24 h after surgery for 14 days.

### 2.2. Surgical Treatment II

One month after the first surgery, radiography revealed bone regeneration at the radial head. Two months following surgery, bone continuity and sufficient size (18.8 mm × 7.5 mm) of the proximal radial head fragment for screw placement were observed. However, loosening of the proximal pins and widening of the ESF pinholes were also noted, leading to the decision that revision surgery was necessary.

**Figure 3:**
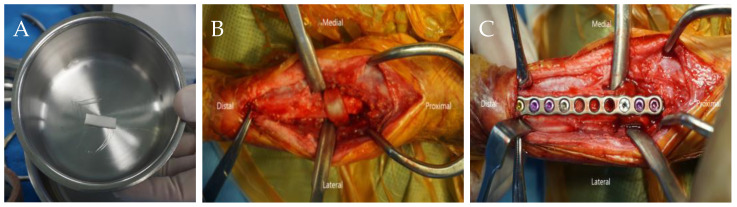
Intraoperative images of the second surgery. (**A**) The collagen membrane loaded with BMP-2; (**B**) a collagen membrane covering the radius defect area; (**C**) locking plate fixation of the radius.

The anesthesia protocol was identical to that used in the previous surgery. A craniomedial incision was made to access the surgical site, elevate the extensor carpi radialis muscle, and separate it from the supinator muscle using a periosteal elevator. In the initial procedure of the second surgery, a segment of the previously implanted bone-substitute material and adjacent bone tissue from the proximal radial head were harvested while trimming the callus that had formed on the radial surface as part of the bone healing process, prior to plate application for internal fixation. A collagen membrane loaded with rhBMP-2 was placed around the defective area to enhance osteogenesis. Fracture fixation was subsequently performed using a locking plate (24-holes 2.0 mm, straight plate, ARIX Vet) (Figure 3A–C). The supinator muscle was then sutured to the surrounding muscles, and the skin layers were closed routinely.

### 2.3. Statistical Data Analysis

All data were analyzed with GraphPad Prism 10 (GraphPad Software, Inc., La Jolla, CA, USA). The HU values of the affected and contralateral radii were compared using the Mann–Whitney U test. The gait analysis data were analyzed with a repeated measures analysis of variance (ANOVA) followed by a post hoc Šídák multiple comparisons test. Statistical significance was defined as *p* < 0.05.

### 2.4. Diagnostic Assessment and Outcomes

One month after the first surgery, radiographic evaluation revealed bone regeneration of the radial head, with maintenance of the ESF and connection of the proximal three fragments. Two months after surgery, the upper radial head formed a single segment (18.8 mm × 7.5 mm), allowing sufficient space for plate fixation, even without hybrid ESF. Therefore, a revision surgery was performed using plate fixation to correct the radius defects and provide a more stable biomechanical environment for the healing bone.

**Figure 4:**
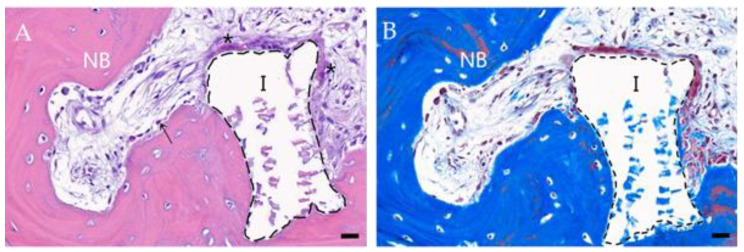
Results of histological analysis at three months after BMP-2 loaded HA and GMP mixture insertion. (**A**) H&E staining, (**B**) Masson’s trichrome staining image showing a section of the bone fragment. I: implant (black dotted lines). NB: new bone. Arrow: osteoblast. Asterisks: osteoclast. Scale bar: 20 μm.

The bone specimens harvested during the revision surgery were histologically processed with hematoxylin and eosin (H&E) and Masson’s trichrome (MT) staining to evaluate the regenerated bone tissue morphology. In histological analysis of bone regeneration, H&E staining revealed newly formed bone tissue around the HA, with osteoblasts and osteoclasts observed at the HA border (Figure 4A). MT staining of the same area revealed that immature bone tissue was stained blue, whereas mature bone tissue rarely stained brick red (Figure 4B).

Two weeks after the second surgery, the ROM of the affected limb’s elbow joint was found to be mildly reduced by 10–20% compared to that of the normal limb. Radiography revealed that the radial head was positioned below the typical location, resulting in decreased joint congruity. However, the overall condition of the joint improved compared to that at the initial visit, and continuity of the bone within the defect areas was observed. Five weeks after the second surgery, partial recovery of the asymmetrical gait was revealed. Nine weeks after the second surgery, the patient’s gait had improved, and the clinical lameness score criteria revealed 8 points out of 27 points.

**Figure 5:**
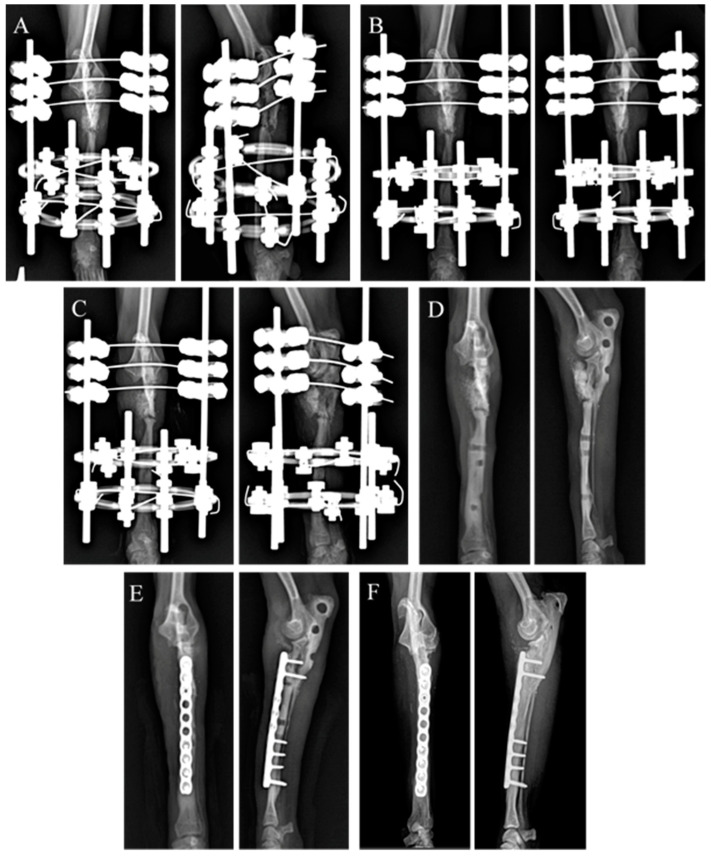
Sequential radiographic images are shown at 0 days (**A**), 4 weeks (**B**), and 8 weeks (**C**) after fixation of the hybrid ESF with bone graft materials. (**D**) Due to ESF pin hole defects, plate fixation with bone graft materials was performed at 12 weeks (**E**). Radiography showing radioulnar synostosis (**F**) 4.8 years after the insertion of the plate.

At the 4.8 years long-term follow-up visit, the owner reported no forelimb-related clinical signs such as lameness. Radiographically, evidence of synostosis between the proximal ulna and radius along with mild osteoarthritis were observed in the affected elbow joint (Figure 5). Furthermore, on CT, while the lengths of both humeri were nearly the same, the right radius was 83.5 mm and the left radius was 70 mm, meaning that the length of the affected limb was approximately 17% shorter than that of the normal limb.

**Figure 6:**
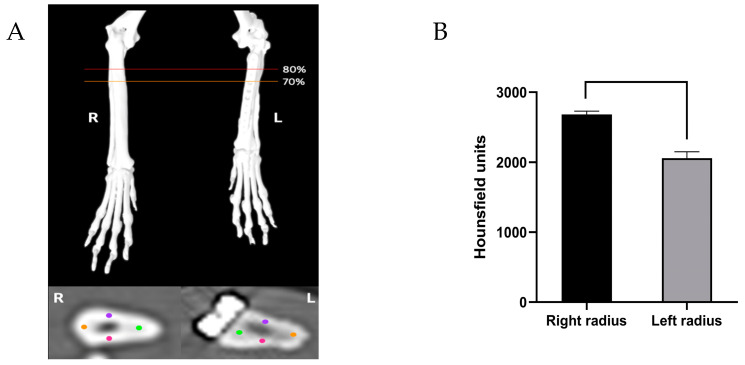
(**A**) Measurement of the Hounsfield unit (HU) values in 70–80% of the distal to proximal length of the affected radius and the same level of the right radius from CT images. Bone mineral density was estimated from HU measurement at the cranial (purple), caudal (pink), medial (green), and lateral (orange) cortices. The mean cross-sectional BMD was calculated. (**B**) A significant difference in the mean HU value between bilateral radii can be observed.

CT was performed to compare and evaluate the bone properties of the contralateral and affected radii (window level, 450 HU; window width, 4500 HU). Based on the 3D reconstructed images of the patient’s normal radius and the surgically treated radius, the Hounsfield unit (HU) value was measured at 70–80% of the distal to proximal length of the affected forelimb where the bone graft was implanted. The cross-sectional areas of the bones were concurrently measured. For the normal radius, the HU measurements were obtained at the same level as those for the affected radius (Figure 6A). In addition, a region-of-interest tool was used to measure the HU at the midpoints of the cortices (C_med_, C_lat_, C_cran_, and C_caud_). The HU values of the normal bone tissue on the right side were significantly higher than those in the area with bone graft insertion (*p* < 0.0001) (Figure 6B).

**Figure 7:**
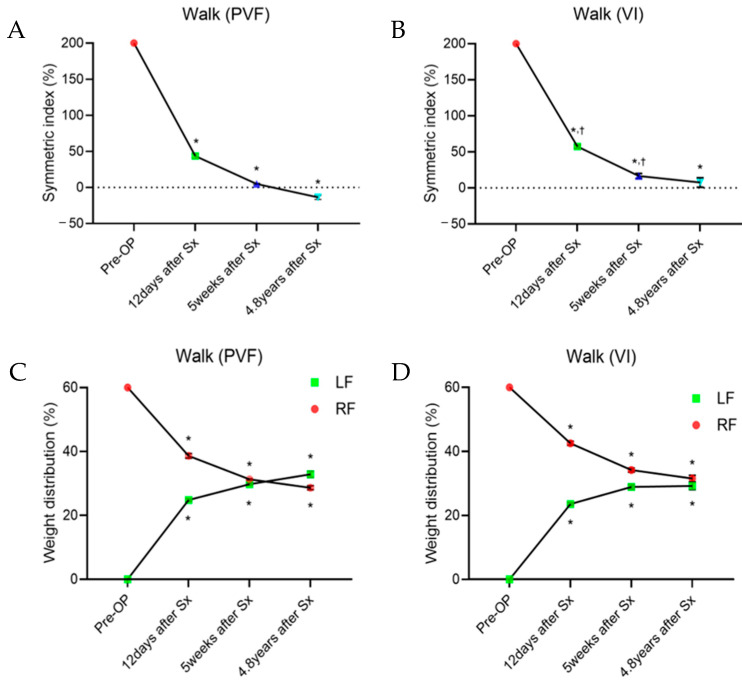
Gait analysis of the pressure sensor walkway. (**A**,**B**) The symmetry index of the left forelimb decreased after fixation of the radius locking plate (2nd Surgery). (**C**,**D**) Weight distribution of the bilateral limbs revealed a tendency towards restoration of the left forelimb function. PVF: peak vertical force; VI: vertical impulse; RF: right forelimb; LF: left forelimb; * significant change from pre-operation; ^†^ significant change from 12 days after fixation of radius locking plate.

The gait analysis after the second surgery was objectively evaluated using a pressure sensor walkway (Walkway, Tekscan Inc., Norwood, MA, USA), and the symmetry index (SI) and weight distribution (WD) were assessed using the data obtained from Tekscan software (Walkway 7.66). The gait analysis results at 12 days, 5 weeks, and 4.8 years post second surgery revealed a reduction in the difference between the peak vertical force (PVF) and vertical impulse (VI) for both forelimbs (Figure 7). Compared with pre-operative values, the SI and WD analysis showed an increase in both PVF and VI in the affected limb (one-way RM ANOVA, *p* < 0.0001). Additionally, the SI-PVF value became negative, indicating an increase in the PVF of the left limb compared with that of the right limb. This suggests an improvement in walking ability at 4.8 years post second surgery.

## 3. Discussion

Stabilization of complex fractures involving small segments near the joint can be challenging. The length and shape of the fractured segment must be carefully considered during the fixation. Otherwise, instances such as very short proximal segments may not provide sufficient space for internal fixation [4,7]. Alternatively, procedures such as radial head ostectomy or elbow arthrodesis are available as salvage interventions [15,16,17,18]. ESF is an effective treatment for the minimally invasive correction and fixation of complex tibia and radius fractures [8]. The Ilizarov method of circular ESF is commonly used for angular limb deformity correction, limb lengthening, and complex juxta-articular fractures. Nevertheless, considering the angle of the elbow joint in a normal standing position, a pure Ilizarov full ring frame may impede elbow movement, which could negatively affect joint mobility during recovery. To minimize interference with elbow flexion, specific rings such as “stretch” or “partial” rings could be used at the proximal part of the frame. However, in the case of juxta-articular fractures of the upper portion of the radius, applying hybrid ESF, which combines the advantages of both linear and circular ESF, may provide a more beneficial solution. Linear ESF offers static axial stiffness for fractures near the joint in the proximal region where screw insertion for plate fixation is challenging. The application of fine-wire fixation to a circular ring further provides strong stability to small bone fragments. Moreover, tensioned wires crossing the ring offer rigid fixation while generating nonlinear dynamic axial micromotion at the fracture site, promoting faster callus formation compared to conventional healing. Furthermore, fixation wires offer multiple fixation points even for extremely small bone fragments [7,10,19]. However, while hybrid ESF provides significant advantages, their overall weight may restrict functional ambulation in underweight patients. Although manufacturers produce systems specifically designed for miniature and small breeds, it is advisable to consider replacing conventional fixators with lighter alternatives, such as acrylic ESF, to maintain comparable biomechanical efficacy while alleviating the mechanical load on low-body-weight patients [4,20]. A hybrid linear-circular ESF can be used to bridge the antebrachium using a hybrid rod attached to the ring, which enables the placement of transfixation pins on the lateral side of the olecranon and extends to the radius and ulna, distal to the fracture site. This method allows the proximal radius to ‘float’, while still being attached to the ulna by ligaments and a joint capsule to maintain its position [4,21]. As the proximal radial region is relatively stable owing to the ulna and ligaments, inserting linear pins into the olecranon and affixing the ring to the distal radius could provide stability. In this case, a hybrid ESF was utilized to preserve the radius without directly stabilizing the fracture site, allowing for temporary floating of the radial head. Although this method aids in maintaining the position of the fractured radial head, the successful union of the fragmented radial head remains uncertain. Consequently, tissue engineering techniques may be considered to enhance bone regeneration and facilitate successful healing.

Bone tissue can regenerate naturally following minor damage without exceeding the critical size threshold. However, substantial defects beyond this threshold require clinical intervention for functional recovery and eventual complete healing [22,23]. The application of bone fixation plates, bone allografts, or autografts is the standard treatment for substantial bone defects [24]. Despite these approaches, concerns regarding the necessity for subsequent removal and the potential side effects persist. The BTE strategy aims to develop materials that promote bone regeneration at defect sites without causing these problems [25]. These biomaterials promote bone regeneration through one or more of the following processes: osteoinduction, osteoconduction, or osteogenesis. In orthopedic defects of load-bearing anatomical sites, such as long bones, the development of new bone tissue must occur quickly to alleviate pain. It is preferable to use osteoconductive materials that provide a three-dimensional structure at the defect site along with osteoinductive materials that induce ossification [26]. In this case, HA and BMP-2 were applied as osteoconductive and osteoinductive materials, respectively (Figure 2). HA (Ca_10_(PO_4_)_6_(OH)_2_) fills the void space at the implant site while providing a three-dimensional structure that allows the growth of the microvasculature, surrounding tissues, and osteoprogenitors into the bone interior [27,28]. BMP-2 stimulates the differentiation and proliferation of mesenchymal stem cells into osteoblasts, thereby promoting bone formation, maturation, and ossification [25]. However, the direct administration of high doses of BMP-2 can lead to side effects, including osteoclast activation and inflammatory responses [14]. Furthermore, as bone healing occurs over several months, it is crucial that BMP-2 be released slowly. Experimental studies [29,30] have demonstrated successful bone regeneration through the utilization of BMP-2-loaded GMP and a collagen membrane, inducing sustained release of BMP-2, thus facilitating osteogenesis. Woven bone with irregular collagen fibers is immature and typically found in developing bones and fracture healing sites. This material is subsequently replaced by lamellar bone, comprising of organized collagen layers [31]. In this case, histological analysis with H&E and MT staining revealed regions of bone matrix containing both irregularly woven and multilayered lamellar bone (Figure 4). The presence of blood vessels and bone cells such as osteoblasts and osteoclasts suggests active bone remodeling, growth, and maintenance in areas where HA is present [28,32]. The observed histological evidence supports the osteoconductive properties of HA and osteoinductive properties of BMP-2 at the fracture site, promoting osteogenesis and bone regeneration.

CT is increasingly recognized as a valuable tool for the noninvasive measurement of bone mineral density, utilizing the HU scale based on the standard linear attenuation coefficient [33,34]. For the quantitative assessment based on CT images, the HU values were measured at the midpoints of the cortices (Figure 6A). In this present case, radioulnar synostosis (Figure 5F), a common complication of radioulnar fracture repair in dogs and cats was observed, resulting in an inconsistent bone shape. CT examination revealed that the affected radius had lower HU values than the opposite forelimb. In addition, the cross-sectional area of the distal left radius, where there was no plate, was larger than the corresponding area of the right radius. Lower HU values and increased cross-sectional areas were observed because the left forelimb was 17% shorter than the right forelimb, as shown in the CT scan. Generally, leg length discrepancy results in shorter leg dragging during walking, leading to increased vertical movement of the center of gravity and, consequently, increased energy consumption [35]. Stress refers to the resistance within bone tissue that develops in response to loads such as compression, bending, and torsion applied to the bone. According to Wolff’s law, when a specific bone is subjected to increased loads, it remodels over time to become stronger and to withstand such loads, resulting in increased bone thickness [34,36]. Bone density depends on the applied load; a decreased load leads to decreased density and can cause osteoporosis over time. When a plate with a high Young’s modulus is used to fix a fractured bone, the load is transferred to the plate, thereby altering the stress distribution. This reduces bone stress, causing stress shielding, which decreases bone density and elasticity, progressively weakening the bone [37,38]. In this case, surgery was performed at the age of 1, a highly active age, allowing the bone to experience a significant load-bearing environment as walking improved postoperatively. To adapt to the increased vertical movement of the center of gravity and the resulting increase in impact, the left radius was speculated to have thickened and strengthened. However, the lower HU values on the left side of the CT scan were attributed to the plate causing less stress to the bone, leading to the activation of osteoclasts that perceived unnecessary bone tissue, resulting in a relative decrease in bone density.

Recently, gait analysis has been increasingly applied to objectively detect and monitor lameness in small animals with musculoskeletal or neurological disorders [39,40]. In this case, kinetic gait analysis based on paw pressure was used to assess postoperative gait. At 12 and 5 weeks after the second surgery (Figure 7), the differences in the PVF and VI between the affected and normal limbs tended to decrease, approaching an SI of zero. At 4.8 years, distinctive gait characteristics of the shorter left limb were observed, including increased vertical displacement of the center of mass and decreased stance time, stride length, and walking speed [35]. The patient also exhibited these characteristics in the shorter left forelimb, resulting in an increased PVF and decreased VI. Consequently, the SI-PVF value becomes negative, whereas the SI-VI value remains positive. At 4.8 years, the absolute mean values of SI-PVF and SI-VI were 3.53% and 11.64%, respectively. Even in healthy dogs, there can be a maximum of <6% asymmetry between limbs [41]. The SI generally records the differences between normal and pathological gaits and evaluates changes in asymmetry due to enhanced pathological conditions [42]. Although the SI-VI value was relatively more asymmetric, owing to the reduced stance time of the left limb compared to that of the normal limb, the SI-PVF fell within the range of asymmetry observed in normal dogs.

This study has several limitations. First, as we presented only a single case, additional prospective studies are required to validate the feasibility and repeatability of the hybrid ESF and BTE techniques in promoting proper bone regeneration. The use of threaded pins, which offer better bone holding power, is recommended for linear ESF applications [43]. Unfortunately, smooth pins were used in this study, and periodic monitoring was conducted to minimize potential instability. Despite the non-ideal pins, BMP-2 was applied to the fracture site, resulting in successful bone healing. Furthermore, fusion of the radius and ulna occurred after the procedure, resulting in impaired physiological joint movements. This complication has also been reported in previous studies [21], highlighting the need for further investigations to prevent such complications. In addition, it is possible to consider removing the screws while checking the bone density through periodic monitoring.

## 4. Conclusions

In conclusion, the patient experienced a radial head fracture during the growth period. Long-term follow-up revealed complications, including decreased joint congruity, radioulnar synostosis, and arthritis development. Despite these issues, joint preservation remains a meaningful outcome. However, it is important to recognize that this is based on a single clinical case and may not be widely applicable without further research. Additionally, the patient’s low body weight likely contributed to an easier adaptation to biomechanics. The successful application of hybrid ESF and BTE techniques to unite complex radial head fractures and defects highlights the potential of this approach as a viable treatment option, prioritizing joint preservation over salvage interventions.

## Data Availability

All relevant data is contained within the article, the original contributions presented in the study are included in the article. Further inquiries can be directed to the corresponding author.
